# Management strategies and outcomes of thromboembolism prevention in atrial fibrillation co-existing with immune thrombocytopenia: A review of evidence

**DOI:** 10.1016/j.ijcha.2024.101493

**Published:** 2024-08-22

**Authors:** Omar H. Metwally, Alaa Rahhal, Raghad A. Elsherif, Ahmed M. Elshoeibi, Mohamed Elhadary, Amgad M. Elshoeibi, Ahmed Badr, Basel Elsayed, Mona Al- Rasheed, Awni Alshurafa, Mohamed A. Yassin

**Affiliations:** a College of Medicine, QU Health, Qatar University, Doha, Qatar; b Pharmacy Department, Heart Hospital, Hamad Medical Corporation (HMC), Doha, Qatar; c School of Medicine, New Giza University, Giza, Egypt; d Hematology Department, AL Adan Hospital, Kuwait City, Kuwait; e Hematology Section, Medical Oncology, National Center for Cancer Care and Research (NCCCR), Hamad Medical Corporation, Doha, Qatar

**Keywords:** Atrial fibrillation, Immune thrombocytopenia, Thromboembolism prevention, Bleeding risks

## Abstract

This review aimed to assess bleeding risks and explore management options in atrial fibrillation (AF) patients with immune thrombocytopenia (ITP), aiming to formulate an optimal therapeutic approach for improved patient prognosis. Employing MeSH terms, a comprehensive search strategy identified articles on bleeding risks and management guidelines in AF combined with ITP. Original research papers were included, while animal studies, reviews, and non-English articles were excluded. From four databases, 1891 articles were initially retrieved, resulting in 10 relevant full-text articles. Eight studies investigated the effectiveness of anticoagulants in managing concurrent AF and ITP, demonstrating reduced bleeding risk and promising outcomes. Two papers explored surgical interventions, particularly left atrial appendage closure, suggesting its safety for AF management in patients with primary hemostatic disorders, including thrombocytopenia. While the pathophysiological mechanisms of AF and ITP remain unclear, anticoagulation regimens exhibited promising reductions in bleeding risks. Larger studies are warranted to enhance understanding and investigate optimal treatments for AF and ITP.

## 1 Introduction

Atrial fibrillation (AF) represents a frequently encountered supraventricular tachyarrhythmia within clinical settings [1]. It is closely associated with increasing age and several cardiac conditions [2] and is marked by discordant atrial activation leading to irregularly irregular ventricular response putting patients at risk of heart failure, thromboembolic events [3], [4], [5], and stroke [6]. These complications arise from AF leading to blood stasis in the left atrial appendage [7] eventually leading to atrial enlargement [8]. AF manifests in three types, paroxysmal, persistent, and permanent [6], which can be detected using an electrocardiogram [9]. Despite ongoing research efforts, the precise pathophysiological mechanisms underlying AF remain incompletely understood [10]. AF is often managed by oral anticoagulation to reduce the risk of the thromboembolism [11], [12], including Non-Vitamin K Oral Anticoagulants (NOACs) or vitamin K antagonists (VKAs) [13], antiarrhythmics, and rate controlling agents, including beta-blockers [10].

Another hematological disorder of interest is immune thrombocytopenia (ITP), which is characterized by a reduction in platelet count due to the formation of autoantibodies targeting platelet antigens [14]. The etiopathogenesis of ITP involves a complex interplay between immune dysregulation and platelet destruction. This condition can manifest either as a primary disease or a secondary condition to various triggers such as Systemic Lupus Erythematosus (SLE) [32], [15], Human Immunodeficiency Virus (HIV), hepatitis C [16], or certain medications. Additionally, ITP exhibits various clinical features across distinct age groups, typically presenting as a self-limiting illness in children and as a chronic condition in adults. Symptoms of ITP can vary amongst cases but most commonly include petechiae, gingival bleeding, ecchymosis, and menorrhagia [17]. ITP is diagnosed primarily by a complete blood count and a blood smear [18] and is managed by corticosteroids and intravenous immunoglobulins (IVIG) in most instances followed by second line therapies, including thrombopoietin receptor agonist (TPO-RA), rituximab, or splenectomy amongst non-responders or corticosteroids dependent [33], [19].

Stroke prevention with oral anticoagulation among patients with ITP carries an increased risk of bleeding in view of thrombocytopenia [17], [19], representing a critical challenge for clinicians in real-world settings as it necessitates achieving a reasonable balance between the risks of thromboembolism and bleeding. Therefore, we conducted this review of evidence to explore the available anticoagulation strategies for stroke prevention in AF co-existing with ITP and determine the clinical implications of these strategies on the bleeding risk.

## 2 Materials and methods

### 2.1 Eligibility criteria

We included original research articles that examined the bleeding risks and management strategies of stroke prevention in AF and ITP till 09/07/2023. Experimental studies, observational studies, case series, and case reports published in English were included. We excluded studies that fell into the following categories: (1) animal studies, (2) reviews or non-original articles, and (3) non-English articles.

### 2.2 Search strategy

We developed our search strategy using Medical Subject Headings (MeSH) terms and irrelevant keywords from article titles and abstracts. To ensure comprehensive literature search, we searched PubMed and Embase, Web of Science, and Scopus databases for terms related to ITP and AF, including “Immune Thrombocytopenia,” “Immune Thrombocytopenic Purpura,” “ITP,” “idiopathic thrombocytopenic purpura,”“ atrial fibrillation,” “ atrial flutter,” and “AFib,” We had no restriction to timeframe. All the studies identified through the search strategy were imported into EndNote, where duplicate articles were removed. The remaining studies were transferred to Rayyan for further screening.

### 2.3 Study selection and data extraction

**Fig. 1:**
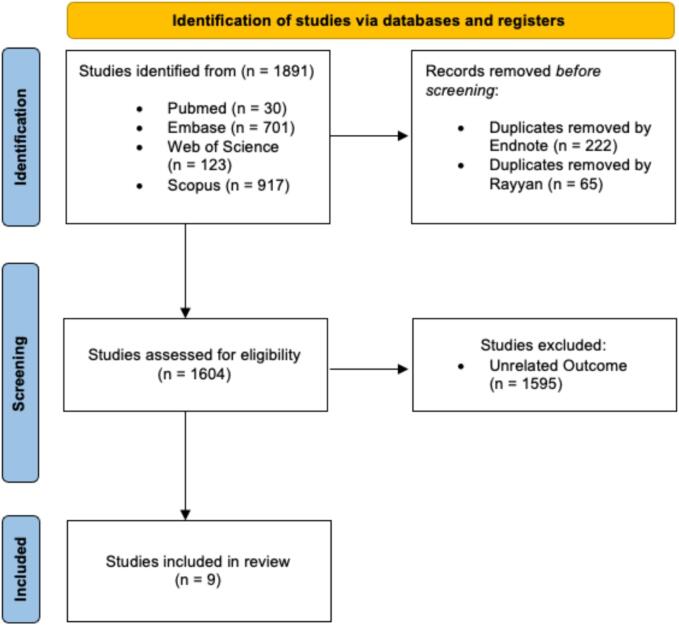
This is a figure. Schemes follow the same formatting.

The titles and abstracts of the records identified were screened by two independent reviewers. Irrelevant records were excluded according to our eligibility criteria as demonstrated in Fig. 1. Then, all relevant abstracts were retrieved in full text and disagreements were resolved through discussions amongst the reviewers. The included articles were tabulated using an Excel sheet/spreadsheet (Table 1) extract the following parameters: article’s last author, year of publication, study design, number of subjects, anticoagulation strategies for AF, baseline platelet count in thrombocytopenia group, and outcomes.

**Table 1:** Showing summary of studies included.

First Author	Publication Year	Study Design	Subjects Number	Anticoagulation Strategies	Baseline platelet count	Outcomes
Janion- Sadowska (20)	2018	Prospective cohort	62	Rivaroxaban, dabigatran, and apixaban (Non-vitamin K antagonist oral anticoagulant)	50 to 100 × 10^9^/Liter	In atrial fibrillation patients with mild thrombocytopenia, Non-vitamin K antagonist oral anticoagulant seems to be effective
Vecchis (21)	2020	Retrospective cohort	220	Edoxaban and enoxaparin sodium	99,000 and 30,000 thrombocytes per mm3	Edoxaban at low doses appears effective for prophylaxis of cardioembolic events atrial fibrillation and thrombocytopenia.
Pastori (22)	2019	Prospective cohort study	5215	One group: Warfarin/acenocoumarol Another group: Dabigatran/apixaban/rivaroxaban/edoxaban	<150 × 10^9^/Litre	Despite an increased incidence of mortality, thrombocytopenia was not associated with mortality at multivariable analysis
Wang (23)	2019	Retrospective Cohort	8239	Warfarin and non-vitamin K antagonist oral anticoagulant (dabigatran, rivaroxaban, apixaban, or edoxaban)	<100 × 10^3^/µLiter	Non-vitamin K antagonist oral anticoagulant therapy is a reasonable choice for stroke prevention in atrial fibrillation patients with thrombocytopenia.
Baldini (24)	2012	Case Report	1	Romiplostim and Warfarin	21x10^9^/Liter	Combination of warfarin and romiplostim was feasible and effective for up to 1 year in a patient with chronic immune thrombocytopenia and severe comorbidities
Cantoni (25)	2012	Case Report	1	Romiplostim	4x10^9^/Litre	Early use of romiplostim is effective and allows the concomitant administration of anticoagulants
Cubero- Gallego (26)	2017	Case Reports	1	eltrombopag	15000/micro litre	Patient developed thrombosis on left atrial appendage occluder even during treatment by eltrombopag
Dognin (27)	2021	Cohort	229	None (percutaneous left atrial appendage closure)	< 100 × 10^9^ cells/Litre	Percutaneous left atrial appendage closure in primary haemostatic disorders carriers appeared as safe and as effective as in overall left atrial appendage closure population for stroke and bleeding prevention at midterm follow-up
Uddin (28)	2021	Case Report	1	None (percutaneous left atrial appendage closure)	70 × 10^9^/Litre	Percutaneous left atrial appendage closure is a good solution for long term anticoagulation to reduce stroke risk, but guidelines are needed for anticoagulation management after the procedure

### 2.4 Objectives and outcomes

The objective of this review to determine available strategies for stroke prevention among patients with AF co-existing with ITP, along with the clinical outcomes of these strategies. The outcomes evaluated were: (1) pharmacological and non-pharmacological strategies for stroke prevention; (2) and the clinical outcomes, including mortality, major bleeding, and thromboembolic events.

## 3 Results

We identified a sum of 1891 articles from the combined results of four databases. These articles were imported into EndNote, where an automated process detected and eliminated 222 duplicate entries. Afterward, the articles were moved to Rayyan, where an additional 65 duplicates were manually identified and eliminated. The process of determining which articles to include or exclude was carried out using Rayyan by two independent reviewers. The remaining 1604 studies were screened for eligibility. Ultimately, 1594 articles were excluded for not meeting the criteria for inclusion, and a total of 10 articles were included in this review as demonstrated in Fig. 1.

The studies included covered the management of AF among patients with ITP as well as complications and risks associated with it as demonstrated in Table 1. Stroke prevention in AF involve the use of anticoagulants, such as VKAs and NOACs, along with surgical management, such as percutaneous left atrial appendage (LAA) closure.

### 3.1 Anticoagulation

A study compared the bleeding complications associated with NOAC use in AF patients with thrombocytopenia versus those with normal platelet counts, using reduced NOAC doses. The incidence of bleeding events was similar between both groups, encompassing minor, major, or clinically relevant nonmajor bleeding. Additionally, there were no significant differences in survival rates, thromboembolic events, major bleeding, or CRNMB between the two groups. However, when major bleeding and CRNMB were combined, the thrombocytopenic group exhibited a significantly higher bleeding risk, as reflected by a higher mean HAS-BLED score compared to the normal platelet count group [20].

The study emphasized that bleeding risk in thrombocytopenia is influenced by various factors, including age, but did not explore other predictors such as unstable blood pressure. Limitations included the modest sample size and the retrospective nature of the data, indicating the need for larger, multicenter prospective trials with extended follow-up periods. Despite these limitations, the study sheds light on the safety and efficacy of reduced NOAC doses in managing AF patients with mild thrombocytopenia, underlining the ongoing need for refined anticoagulation strategies in diverse AF patient populations.

A retrospective cohort study compared low-dose edoxaban (30 mg/day) with enoxaparin in AF patients with moderate thrombocytopenia (platelet count 30–99 × 109/L). The study found that patients on edoxaban had a significantly lower risk of the composite endpoint (all-cause death, transient ischemic attack/stroke, hospitalizations, and major bleeding events) compared to those on enoxaparin, with a hazard ratio of 0.071 (95 % CI: 0.013 to 0.373; p = 0.0019). This protective effect of edoxaban was notably stronger than other variables studied, including hypertension. However, the study notes the need for further investigation into bleeding events related to variceal rupture in liver cirrhosis, which might influence outcomes. The protective benefits of low-dose edoxaban are highlighted, but understanding the dose–response relationship in thrombocytopenic AF patients remains a knowledge gap requiring deeper exploration. The study's retrospective design and confounding by indication are acknowledged limitations, emphasizing the necessity for prospective studies to clarify the observed associations and guide clinical decision-making [21].

A study examined the impact of thrombocytopenia (platelet count < 150 × 109/L) on major bleeding and mortality among atrial fibrillation (AF) patients receiving VKAs or NOACs. Mortality was notably higher in the thrombocytopenia group, especially with moderate-severe thrombocytopenia (<100 × 109/L). This group also had a higher risk of major bleeding. The study revealed disparities in thrombocytopenia prevalence across demographics, such as higher rates in Korean patients and lower rates in white patients. Comorbidities like chronic kidney disease and liver cirrhosis showed associations with thrombocytopenia, urging further research, especially in AF patients. The accelerated decline in platelet count with age in men versus women also warrants deeper exploration. Limitations include the study's population specificity to Italians and lack of information on anticoagulant efficacy in VKA-treated patients. Overall, the study enhances our knowledge but underscores the need for broader investigations into thrombocytopenia dynamics in AF contexts [22].

A study compared the efficacy and safety of NOACs versus warfarin in patients with atrial fibrillation (AF) and thrombocytopenia (platelet count < 100 × 109/L) versus those without thrombocytopenia. NOAC therapy showed a lower trend for major bleeding compared to warfarin but had similar risks of death and ischemic stroke/systemic embolism (IS/SE). This research fills a significant gap by directly comparing anticoagulant therapies in a substantial sample of AF patients with thrombocytopenia, a group often excluded in prior studies. However, limitations such as a retrospective design and focus on a specific population (Taiwanese) suggest the need for broader, prospective studies to confirm these findings across diverse demographics. While NOACs showed promise in reducing major bleeding, ongoing research is crucial to develop precise guidelines for managing AF patients with thrombocytopenia effectively [23]

A 68-year-old man with chronic immune thrombocytopenia (ITP), dilated cardiomyopathy, and atrial fibrillation (AF) was effectively managed using romiplostim for ITP and warfarin due to left atrial appendage thrombosis. The successful combination of thrombopoietin receptor agonists (TRAs) like romiplostim with warfarin offers a promising therapeutic strategy for ITP patients needing ongoing anticoagulation, especially those unsuitable for standard treatments or with inadequate responses to typical therapies. However, despite providing valuable insights, the retrospective nature of the study and the patient's complex comorbidities highlight the need for larger, prospective trials to establish the safety and efficacy definitively. While this case showcases a potential avenue for managing chronic ITP alongside anticoagulation, further research is crucial to develop evidence-based guidelines for such complex clinical scenarios [24].

A 91-year-old man with atrial fibrillation developed immune thrombocytopenia (ITP) while on phenprocoumon anticoagulation, presenting with bleeding symptoms despite initial prednisolone treatment. Due to uncontrolled INR and ongoing bleeding, IVIG, platelet transfusions, and romiplostim (a TPO-RA) were sequentially administered. Romiplostim use led to a steady rise in platelet count without complications. This case suggests that early initiation of romiplostim as a second-line therapy in newly diagnosed, anticoagulated ITP patients can be effective and safe, warranting further investigation for optimal management strategies. Limitations include the lack of consensus on timing and the study's retrospective, single-case nature, necessitating broader studies for conclusive recommendations [25].

A 77-year-old man with atrial fibrillation, diabetes mellitus, hypertension, moderate-to-severe aortic stenosis, and refractory immune thrombocytopenia (ITP) underwent left atrial appendage (LAA) occlusion due to a high bleeding risk on chronic oral anticoagulation. Despite subsequent treatment with warfarin and aspirin, his platelet count remained low, and echocardiography revealed a large adherent thrombus on the LAA occluder. Given his need for long-term treatment with the TPO receptor agonist eltrombopag, the patient underwent surgical LAA closure and bioprosthetic aortic valve replacement. This case underscores the rare occurrence of early thrombosis in LAA occlusion associated with TPO receptor agonist therapy, necessitating surgical intervention. This report emphasizes the limited understanding of using TPO receptor agonists in atrial fibrillation patients undergoing LAA occlusion, highlighting potential safety concerns and the need for further research. The interaction between TPO receptor agonists and device thrombogenicity in LAA occlusion warrants detailed investigation, especially in patients with refractory ITP. This case underscores the complexities and risks involved in managing such patients, urging comprehensive risk assessments and a multidisciplinary approach to guide clinical decisions and refine treatment strategies. Future research is crucial to address these gaps and improve outcomes in similar clinical scenarios [26].

### 3.2 Left atrial appendage closure

A study investigated percutaneous left atrial appendage closure (LAAC) in patients with primary hemostasis disorders (HD) and atrial fibrillation (AF), including 17 patients with HD, of which 5 had thrombocytopenia. The procedural success rate was comparable between HD (100 %) and non-HD (94 %) groups, although HD patients had a lower baseline history of bleeding (53 % vs. 91 % in non-HD). This study advances understanding of LAAC as an alternative to anticoagulant therapy in this population, highlighting thrombocytopenia as the main HD in non-valvular AF. It addresses controversies regarding risk assessment tools like CHA2DS2-VASC and HAS-BLED scores in these patients, emphasizing the need for personalized risk evaluation. However, the study's single-center design and limited sample size warrant larger studies with longer follow-ups to validate outcomes and assess long-term safety and efficacy of LAAC in primary HD and AF. In summary, while promising, further research is essential to establish the enduring benefits and risks of percutaneous LAAC in this specific patient group [27].

A 69-year-old man with multiple comorbidities including hypertension, COPD, diabetes, atrial fibrillation, chronic thrombocytopenia, and recurrent gastrointestinal bleeding underwent elective placement of a WATCHMAN device for left atrial appendage occlusion. Post-procedure, he was started on warfarin and aspirin but developed worsening thrombocytopenia and bleeding, prompting warfarin discontinuation. A follow-up TEE on day 45 revealed a well-seated device but with a thrombus in the LAA. This case highlights LAA closure as an alternative to long-term anticoagulation in stroke prevention but notes challenges in managing device-associated thrombosis, especially in thrombocytopenic patients with AF. The lack of guidelines for this population underscores the need for further research to optimize anticoagulation strategies and develop standardized protocols for better outcomes [28].

## 4 Discussion

The primary aim of this review was to investigate bleeding risks, and management strategies for patients presenting with concurrent AF and ITP. Although the literature pertaining to this topic was scarce, included studies in this review showed the effectiveness of several management strategies, including the use of oral Vitamin K antagonists, NOACs, LAA occlusion [29], in cases of AF co-existing with ITP. The studies have also included a detailed review of the cardiac and non-cardiac adverse effects associated with these aforementioned drugs.

According to the evidence in this review, certain anticoagulation regimens, such as apixaban [30] have been shown to be safe and effective when utilized in patients which coexisting AF and thrombocytopenia, in terms of bleeding risks, such as mitigated thromboembolic events and enhanced platelet count [31]. In contrast, it should be noted that other drugs from the same class, for example, betrixaban [29], did not receive approval from the FDA for use in this particular patient population, underscoring the need for cautious drug selection within this class to avoid unfavorable outcomes.

The scarcity of studies that focused on the interplay of AF and ITP made it necessary to include some papers that were retrospective in nature, which makes it difficult to establish causality [11], however, contained important and beneficial management outcomes. In addition, several of the reviewed studies cannot be generalized to broader populations, as they were conducted in specific countries with certain patient demographics, such as a study that was conducted in Taiwan. Consequently, there arises a need to conduct more extensive investigations on this subject matter by conducting studies with larger sample sizes and across various patient cohorts, which will draw more definitive and generalizable conclusions. By conducting more research, we will obtain a more robust understanding of the mechanism of atrial fibrillation and immune thrombocytopenia as dangerous conditions on their own, as well as understand the best approach to managing patients who suffer from both conditions concomitantly which will eventually improve its prognosis.

## 5 Limitations

This review of evidence answered a crucial clinical question that represents a challenge to clinicians in practice. However, in view of scarcity of evidence, case reports were included in the review, which have limited the possibility of combining the outcomes of interest to draw robust conclusions.

Additionally, a few studies included in this review were conducted in one country which also reduces the ability to generalize the results to other populations. Furthermore, many of the studies had a small sample size which can also affect the generalizability of the results and the ability to form subgroups and investigate specific interactions between them.

## 6 Conclusions

In summary, this review focused on exploring the risk of bleeding associated with AF in patients with ITP and examining the available management strategies. While the precise pathophysiological mechanisms underlying atrial fibrillation and ITP remain incompletely understood, the review highlighted the complex interplay between thromboembolic risk and bleeding complications in this specific patient population.This review examines the bleeding risks associated with atrial fibrillation (AF) in patients with immune thrombocytopenia (ITP) and evaluates current management strategies. While the exact mechanisms of AF and ITP are not fully understood, the review highlights the intricate balance between thromboembolic risk and bleeding complications in these patients.

Key gaps in research include the safety and efficacy of non-vitamin K oral anticoagulants (NOACs) in those with low platelet counts, as they were excluded from major trials. There is a need for guidelines on anticoagulation therapy, especially regarding dosing and management after left atrial appendage (LAA) occlusion. Additionally, the relationship between platelet count, antithrombotic therapy, and device-associated thrombosis (DAT) is not well understood. Other areas needing exploration include NOAC-induced thrombocytopenia, long-term safety of thrombopoietin receptor agonists (TPO-RAs), and the impact of comorbidities like liver cirrhosis. Prospective studies and clinical trials are essential to develop evidence-based protocols and risk stratification models for managing these complex cases effectively.The findings of this review suggest that several anticoagulation regimens have shown promise in terms of safety and effectiveness when used in patients with AF and thrombocytopenia. However, additional research studies with larger sample sizes and diverse populations need to be conducted to advance our understanding of the relationship between atrial fibrillation AF and ITP. These studies will help draw more definitive conclusions and provide valuable insights into optimal anticoagulation approaches and alternative therapies for patients with AF and ITP.

## 7 Clinical significance

This research is clinically significant as it addresses the challenges in managing atrial fibrillation (AF) patients with immune thrombocytopenia (ITP). AF requires anticoagulation to prevent strokes, but ITP increases bleeding risks. The review emphasizes effective anticoagulant therapies in reducing bleeding risks in this specific patient group and explores surgical interventions like left atrial appendage closure. It underscores the need for tailored treatment guidelines and larger studies to optimize outcomes for these patients, balancing stroke prevention with bleeding risks. Overall, it guides clinicians in making informed decisions for better patient outcomes in AF with ITP.

## 8 Use of AI tools

AI tools were used to improve language and readability.

## Funding

Open Access funding was generously provided by the Qatar National Library.

## CRediT authorship contribution statement

**Omar H. Metwally:** Writing – review & editing, Writing – original draft, Visualization, Validation, Supervision, Software, Resources, Project administration, Methodology, Investigation, Funding acquisition, Formal analysis, Data curation, Conceptualization. **Alaa Rahhal:** Writing – review & editing, Writing – original draft, Conceptualization. **Raghad A. Elsherif:** Writing – review & editing, Writing – original draft. **Ahmed M. Elshoeibi:** Writing – review & editing, Writing – original draft. **Mohamed Elhadary:** Writing – review & editing, Writing – original draft. **Amgad M. Elshoeibi:** Writing – review & editing, Writing – original draft. **Ahmed Badr:** Writing – review & editing, Writing – original draft. **Basel Elsayed:** Writing – review & editing, Writing – original draft. **Mona Al- Rasheed:** Writing – review & editing, Conceptualization. **Awni Alshurafa:** Writing – review & editing, Conceptualization. **Mohamed A. Yassin:** Writing – review & editing, Writing – original draft, Conceptualization.

## Declaration of competing interest

The authors declare that they have no known competing financial interests or personal relationships that could have appeared to influence the work reported in this paper.

## References

[1] Troughton R.W., Asher C.R., Klein A.L. (2003). The role of echocardiography in atrial fibrillation and cardioversion. Heart.

[2] Wasmer K., Eckardt L., Breithardt G. (2017). Predisposing factors for atrial fibrillation in the elderly. J. Geriatr Cardiol.

[3] Benjamin E.J. (1998). Impact of atrial fibrillation on the risk of death: the Framingham heart study. Circulation.

[4] Chugh S.S. (2001). Epidemiology and natural history of atrial fibrillation: clinical implications. J. Am Coll. Cardiol.

[5] Patel N.J. (2014). Contemporary trends of hospitalization for atrial fibrillation in the United States, 2000 through 2010: implications for healthcare planning. Circulation.

[6] Waktare J.E. (2002). Cardiology patient page. Atrial fibrillation. Circulation.

[7] Giancaterino S., Hsu J.C. (2019). Valvular atrial fibrillation: a confusing and obsolete definition. J. Am. Coll. Cardiol..

[8] Sanfilippo A.J. (1990). Atrial enlargement as a consequence of atrial fibrillation. A Prospective echocardiographic study. Circulation.

[9] Soliman E.Z., Bhave P.D., Chen L.Y. (2019). Electrocardiographic diagnosis of atrial arrhythmias. JAMA.

[10] Hindricks G. (2021). 2020 ESC Guidelines for the diagnosis and management of atrial fibrillation developed in collaboration with the European Association for Cardio-Thoracic Surgery (EACTS): The Task Force for the diagnosis and management of atrial fibrillation of the European Society of Cardiology (ESC) Developed with the special contribution of the European Heart Rhythm Association (EHRA) of the ESC. Eur. Heart J..

[11] Lip G.Y.H. (2018). Antithrombotic therapy for atrial fibrillation: CHEST guideline and expert panel report. Chest.

[12] Gutierrez C., Blanchard D.G. (2016). Diagnosis and treatment of atrial fibrillation. Am Fam. Physician.

[13] Kirchhof P. (2016). 2016 ESC Guidelines for the management of atrial fibrillation developed in collaboration with EACTS. Eur. Heart J..

[14] Rodeghiero F. (2009). Standardization of terminology, definitions and outcome criteria in immune thrombocytopenic purpura of adults and children: report from an international working group. Blood.

[15] Segal J.B., Powe N.R. (2006). Prevalence of immune thrombocytopenia: analyses of administrative data. J. Thromb Haemost.

[16] Kistangari G., McCrae K.R. (2013). Immune thrombocytopenia. Hematol Oncol Clin. North Am.

[17] Izak M., Bussel J.B. (2014). Management of thrombocytopenia. F1000Prime Rep.

[18] Neunert C. (2011). The American Society of Hematology 2011 evidence-based practice guideline for immune thrombocytopenia. Blood.

[19] Provan D. (2019). Updated international consensus report on the investigation and management of primary immune thrombocytopenia. Blood Adv..

[20] Janion-Sadowska A. (2018). Non-Vitamin K Antagonist Oral Anticoagulants in Patients With Atrial Fibrillation and Thrombocytopenia. J. Cardiovasc Pharmacol.

[21] Vecchis R., Paccone A., Soreca S. (2020). Thrombocytopenia-related problems in patients with concomitant atrial fibrillation requiring antithrombotic prevention: a retrospective cohort study. Arq. Bras. Cardiol..

[22] Pastori D. (2019). Thrombocytopenia and mortality risk in patients with atrial fibrillation: an analysis from the START registry. J. Am. Heart Assoc..

[23] Wang C.L. (2019). Effectiveness and safety of non-vitamin-K antagonist oral anticoagulants versus warfarin in atrial fibrillation patients with thrombocytopenia. J. Thromb Thrombolysis.

[24] Baldini S. (2013). Long-term follow-up of concomitant treatment with romiplostim and warfarin in a patient with immune thrombocytopenia and severe cardiac comorbidities. Platelets.

[25] Cantoni N., Heizmann M., Bargetzi M. (2012). Immune thrombocytopenia and anticoagulation: the role of romiplostim in the early treatment. Br. J. Haematol..

[26] Cubero-Gallego H. (2018). Thrombosis of a left atrial appendage occluder after treatment with thrombopoietin receptor agonists. JACC Cardiovasc. Interv..

[27] Dognin N. (2022). Percutaneous left atrial appendage closure in patients with primary hemostasis disorders and atrial fibrillation. J. Interv Card Electrophysiol.

[28] Uddin M.M. (2021). Challenges of left atrial appendage closure device and anticoagulation in a patient with immune thrombocytopenia (ITP). BMJ Case Rep.

[29] January C.T. (2019). 2019 AHA/ACC/HRS Focused Update of the 2014 AHA/ACC/HRS guideline for the management of patients with atrial fibrillation: a report of the american college of cardiology/american heart association task force on clinical practice guidelines and the heart rhythm society. J. Am. Coll. Cardiol..

[30] Page R.L. (2016). 2015 ACC/AHA/HRS guideline for the management of adult patients with supraventricular tachycardia: a report of the american college of cardiology/american heart association task force on clinical practice guidelines and the heart rhythm society. Circulation.

[31] Wang T.Y. (2019). Effectively initiating and maintaining anticoagulation in patients with atrial fibrillation. Circulation.

[32] Ali E.A., Rasheed M., Al-Sadi A., Awadelkarim A.M., Saad E.A., Yassin M.A. (2022). Immune Thrombocytopenic Purpura and Paradoxical Thrombosis: A Systematic Review of Case Reports. Cureus.

[33] Rahhal A., Provan D., Ghanima W., González-López T.J., Shunnar K., Najim M., Ahmed A.O., Rozi W., Arabi A., Yassin M. (2024). A practical guide to the management of immune thrombocytopenia co-existing with acute coronary syndrome. Frontiers in medicine.

